# Shape Sensing of a Complex Aeronautical Structure with Inverse Finite Element Method

**DOI:** 10.3390/s21041388

**Published:** 2021-02-17

**Authors:** Daniele Oboe, Luca Colombo, Claudio Sbarufatti, Marco Giglio

**Affiliations:** Mechanical Engineering Department, Politecnico di Milano, Via La Masa 1, 20156 Milano, Italy; luca1.colombo@polimi.it (L.C.); claudio.sbarufatti@polimi.it (C.S.); marco.giglio@polimi.it (M.G.)

**Keywords:** inverse Finite Element Method, iFEM, shape sensing, optical fiber, aeronautical structure, superimposition of the effects

## Abstract

The inverse Finite Element Method (iFEM) is receiving more attention for shape sensing due to its independence from the material properties and the external load. However, a proper definition of the model geometry with its boundary conditions is required, together with the acquisition of the structure’s strain field with optimized sensor networks. The iFEM model definition is not trivial in the case of complex structures, in particular, if sensors are not applied on the whole structure allowing just a partial definition of the input strain field. To overcome this issue, this research proposes a simplified iFEM model in which the geometrical complexity is reduced and boundary conditions are tuned with the superimposition of the effects to behave as the real structure. The procedure is assessed for a complex aeronautical structure, where the reference displacement field is first computed in a numerical framework with input strains coming from a direct finite element analysis, confirming the effectiveness of the iFEM based on a simplified geometry. Finally, the model is fed with experimentally acquired strain measurements and the performance of the method is assessed in presence of a high level of uncertainty.

## 1. Introduction

Structure monitoring has received a lot of interest in the last decades, both from research institutes and industries. Relevant parameters are measured on the structure to assess its health condition with Structural Health Monitoring (SHM) approaches, to early predict failures and provide condition-based or predictive maintenance, decreasing the operative costs [1]. 

Among the different monitoring approaches available in the literature, displacement monitoring, also known as shape sensing, is receiving more attention especially for usage monitoring [2], although some extensions to damage isolation are present in the literature [3]. Many sensor technologies can be exploited to measure the displacement field, leveraging both on direct and inverse procedures. The first category is related to a direct measurement of the displacement field, e.g., with lasers [4,5] and optical cameras [6,7,8]; however, these can be practically exploited only on laboratory tests and on fixed structures like bridges. Dealing with aeronautical structures, weight and space saving is the main concern, limiting the adoption of the previously cited technologies. In this case, shape sensing can be performed with inverse algorithms, computing the displacement field from strain measurements acquired on the structure itself. Several approaches are available in the literature which can be classified into four main categories according to their working principle [9]: (1) Numerical integration of experimental strains [10,11]; (2) adoption of continuum functions to approximate the displacement field [12,13,14]; (3) Artificial Neural Networks (ANN) [15,16]; (4) method based on finite-elements discrete variation principles [17,18,19]. Among the latter, the inverse Finite Element Method (iFEM) offers several advantages, including the independence from material properties and loading conditions, the lack of training procedures, and the limited computational demand, which makes it suitable for real-time applications. Furthermore, the iFEM has been recently compared with other shape sensing algorithms, like the Modal Method and Ko’s displacement theory, highlighting its accuracy and versatility [20].

The iFEM is a model-based technique originally developed by Tessler et al. [21,22] for the structure’s displacement field computation based on local strain measurements. In particular, only a mesh discretization of the structure with its boundary conditions and strain measurements are required as input to the algorithm, while no information regarding load and material properties is needed, which makes the algorithm attractive for structures subjected to variable and unknown load history, such as for many aeronautical components. The iFEM is based on the minimization of a least-square error function comparing the input strain field acquired by sensors and a numerical formulation of the same, as a function of the nodal degree of freedoms (dofs). Once the nodal dofs (i.e., the displacement field) have been computed, strains are defined as in any direct finite element analysis [23,24]. Different iFEM’s formulations are available in the literature for beam [18,19,25,26,27] and shell-like structures, covering most of the engineering applications. In particular, focusing on shells, three main types of elements have been developed: (i) An inverse three nodes element based on bilinear anisoparametric shape functions, namely iMIN3 [17,28]; (ii) the iQS4, a four nodes flat element with bilinear anisoparametric shape functions [29,30,31]; and (iii) the curved eight nodes iCS8 element with quadratic isoparametric shape functions [32]. These have been recently assessed with each other on a comparative study [33], demonstrating how the iQS4 is more accurate than the iMIN3 for plane problems, while, in the case of curved structures, the best accuracy is archived by the iCS8. Then, other formulations exploit a combined use of iFEM and the Refined Zig-Zag theory (RZT) to compute the through-the-thickness displacement field on composite structures [34,35], while, the combination of isogeometric analysis and iFEM is beneficial for large non-linear deformations [36]. Finally, the iFEM was also recently extended to damage identification [30,37,38,39] by considering that discrepancies between the physical structure and the iFEM model generate wrong displacement and strain field reconstructions.

Several shape sensing applications with iFEM are reported in the literature. However, these are mainly referred to simple plates or structures representative of wing profiles with limited experimental validation. The authors want to extend the iFEM applicability to real structures, proposing a combined use of the superimposition of the effects with non-trivial geometrical simplifications on a complex aeronautical structure. The whole procedure is assessed with both a numerical and experimental application, assessing the iFEM strain reconstruction also far from the input sensors and in presence of complex displacement and strain fields. In particular, limitations on the number and locations of input sensors, acquired by a LUNA ODISI-B system, impose different model simplifications, which are compensated by the linear superimposition of two different iFEM models, as already proposed by the authors in [37], accounting for the actual stiffness of the boundary constraints. Furthermore, the adopted sensor technology limits the acquisition to a single strain component at each measurement location, resulting in a partial definition of the input strain field, where most of the measures are aligned with the load direction, few measurements are transversal to the load direction, and no measurements are available for the shear strain component. Thus, the model contains only rectangular elements aligned with the load direction to avoid numerical errors in the change of reference system from global to local coordinates. Finally, strains are pre-extrapolated with the Smoothing Element Analysis [35,40,41,42] where physical sensors are not available, increasing the overall reconstruction’s accuracy. The overall procedure is first assessed with a numerical study, where the structure is modeled with a direct finite element analysis to compute the reference displacement field for a specific loading condition. Then, strains are numerically computed from the direct FEM to fed different iFEM models and assess their shape sensing capability. Finally, the iFEM model is tested with experimentally acquired strain measurements to verify the shape and strain sensing capabilities in a realistic application.

This paper is structured as follows. The general iFEM framework is briefly described in Section 2, then the aeronautical structure and the experimental test rig are presented in Section 3. Section 4 describes the different models exploited, for both direct FEM and iFEM, while results and discussion are reported in Section 5. Finally, the conclusions are summarized in Section 6.

## 2. Inverse Finite Element Method

A brief description of the inverse FEM is reported in this section since a detailed review is available in [29,30] for the interested readers.

Considering a mechanical shell structure with a given set of boundary conditions and a pattern of strain sensors, the iFEM computes the full displacements field by means of strain–displacement relationships. It is a mesh-based algorithm in which the structure is discretized into inverse finite elements. In particular, the four-node iQS4 shell elements will be analyzed hereafter. The formulation of each inverse element is based on the minimization of a least-square functional defined as the error between the input strain field $\left(\cdot^{\varepsilon }\right)$ and its numerical formulation (⋅(u)), which is a function of the nodal degree of freedoms (dof) u. The error functional of the *i*-th inverse element is defined as:

(1) $$\Phi_{i}\left(u^{i}\right)=w_{m}\|e\left(u^{i}\right)-e_{i}^{\varepsilon }{\|}^{2}+w_{b}\|k\left(u^{i}\right)-k_{i}^{\varepsilon }{\|}^{2}+w_{s}\|g\left(u^{i}\right)-g_{i}^{\varepsilon }{\|}^{2}$$

The strain field is decoupled into three main contributions: (i) The membrane strain component e, (ii) the bending strain component k, and (iii) the transverse shear strain component g. The coefficients $w_{m}$, $w_{b}$, and $w_{s}$ are positive weighting coefficients associated with the related strain components, controlling the coherence between the input and the numerical strain field.

### 2.1. Numerical Strain Formulation

**Figure 1:**
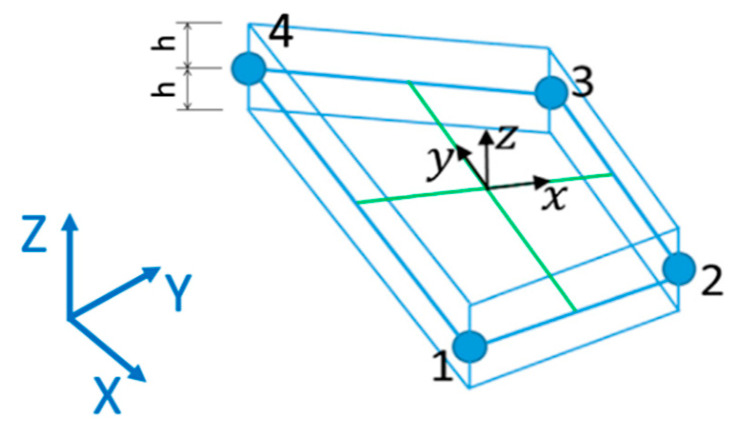
iQS4 element with local, (*x*, *y*, *z*), and global, (*X*, *Y*, *Z*), reference system.

The numerical strain field previously introduced in Equation (1) is based on bilinear shape functions originally developed for the MIN4 element [43,44]. This is referred to a local reference system (x, y, z) centered in the centroid of each inverse element and with z∈[−h;+h], as described in Figure 1. Each iQS4 element has 24 dof, 3 translations, and 3 rotations per node, which are defined in the local reference system within the element. Thus, a proper transformation matrix is required to link the displacement field in global and local coordinates, as detailed in Appendix A. Finally, after some mathematical manipulations, the matrices $B^{m}$, $B^{b}$, $B^{s}$, containing the partial derivatives of the shape functions, link the element’s dof in local coordinates with the numerical strain field.

(2) $$e\left(u^{i}\right)=B^{m}u^{i}\;k\left(u^{i}\right)=B^{b}u^{i}g\left(u^{i}\right)=B^{s}u^{i}$$

### 2.2. Input Strain Formulation

**Figure 2:**
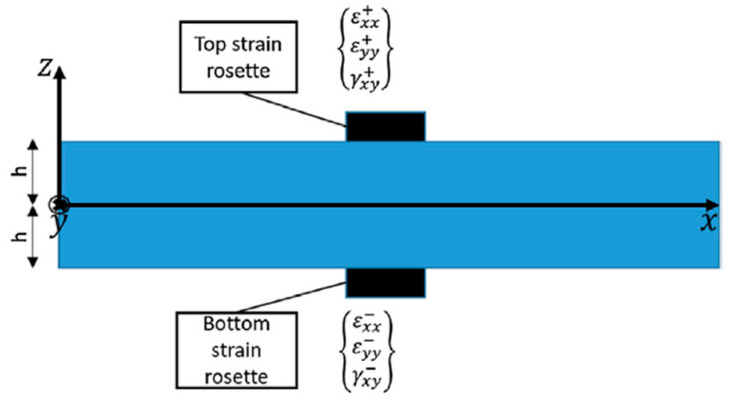
Discrete sensor location on the shell structure.

Considering a strain sensor network installed on the structure, sensors’ data must be elaborated into the three input strain field components included in Equation (1). To correctly decouple the membrane strain field from the bending strain contribution, in all the shell’s spatial directions, a strain gauge rosette can be applied at the same location on both sides of the shell, as graphically described in Figure 2. The mid-plane membrane and the bending contributions of the *j*-th sensor located on the *i*-th inverse element can be computed as:

(3) $$e_{i,j}^{\varepsilon }=\frac{1}{2}{\left\{\begin{array}{c} \varepsilon_{xx}^{+}+\varepsilon_{xx}^{-}\\ \varepsilon_{yy}^{+}+\varepsilon_{yy}^{-}\\ \varepsilon_{xy}^{+}+\varepsilon_{xy}^{-} \end{array}\right\}}_{j}\;\;\;\;k_{i,j}^{\varepsilon }=\frac{1}{2h}{\left\{\begin{array}{c} \varepsilon_{xx}^{+}-\varepsilon_{xx}^{-}\\ \varepsilon_{yy}^{+}-\varepsilon_{yy}^{-}\\ \varepsilon_{xy}^{+}-\varepsilon_{xy}^{-} \end{array}\right\}}_{j}$$

where 2h is the shell’s thickness on the sensor’s location considered, $\left(\cdot^{+}\right)$ the strain measure associated with the sensor on the top side of the shell, and $\left(\cdot^{-}\right)$ the strain measure acquired on the bottom side of the shell.

The transverse shear component g cannot be directly computed from the plain-strain field acquired by surface sensors, however, its contribution is negligible for thin shells.

Equation (3) allows the input strain field definition on the whole model considering strains obtained from physical or virtual sensors, in the latter case leveraging on a simulated environment with direct FE analysis. However, when dealing with experimental tests, the cost of sensors, acquisition system limitations and physical constraints often limit sensor installation on the whole structure, preventing the full input strain field definition. In this case, the elements free from any sensor can leverage on pre-extrapolated strain measurements, for example, exploiting the Smoothing Element Analysis (SEA) [40,41,45,46,47,48]. However, since pre-extrapolated strains are reasonably less accurate than sensor’s strains, a small weighting coefficient $w_{\left(\cdot \right)}$ will be associated to these elements (e.g., $10^{-5}$), while unitary value is assumed for elements including physical sensors.

In general, the sensors’ strain field is conventionally defined in the global reference system (X, Y, Z) by the operator, thus, a transformation matrix $T^{i}$ is applied for its definition in the local reference system and to introduce strain components in Equation (1). Considering a 3D strain tensor ${\overset{=}{\varepsilon }}_{g}$ in the global reference system, on which the sensors’ input plane-strain field is defined, its transformation in the local reference system can be obtained with:

(4) $${\overset{=}{\varepsilon }}_{l}=T^{i}\cdot {\overset{=}{\varepsilon }}_{g}\cdot T^{i^{T}}$$

where ${\overset{=}{\varepsilon }}_{l}$ is the strain field defined in the local reference system and the transformation’s matrix formulation is detailed in Appendix A. After the computation of the strain field in local coordinates for the top and bottom side sensors with Equation (4), the membrane and the bending strain components can be defined directly in local coordinates with Equation (3).

**Figure 3:**
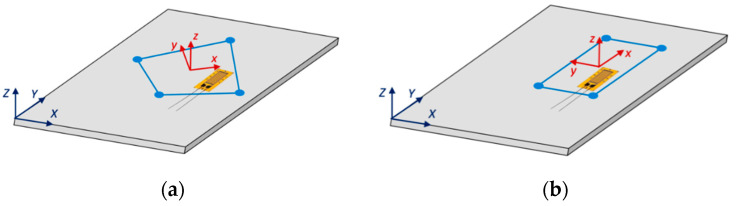
Strain transformation from global to element’s local reference system: (**a**) Element with an arbitrary shape and orientation; (**b**) rectangular element aligned with the strain gauge rosette.

In the most general case, only if a full plane-strain field (i.e., $\varepsilon_{XX}$, $\varepsilon_{YY}$, and $\varepsilon_{XY}$) is correctly acquired by input sensors, the transformation will lead to correct results, otherwise, errors may be introduced. For example, consider an inverse element covered by a monoaxial strain sensor aligned with the *Y*-axis in the global reference system, as reported in Figure 3. The input strain field in global coordinates is represented by a tensor in which $\varepsilon_{YY}$ corresponds to the sensor’s measure and all the other terms are null since they are not measured. Once the transformation matrix is applied, the local strain field may include a full plane-strain definition, according to the element’s geometry. In addition, strain components associated with sensor’s measure are associated with a different weighting coefficient $w_{\left(\cdot \right)}$ than measureless or pre-extrapolated components, which cannot be correctly assessed after the transformation, and thus introducing some errors. This can be observed in the example of Figure 3a, where the resulting strain field in local coordinates contains non-null values in all plain directions and the result is based on a partial definition of the input field, as also detailed in the following equation.

(5) $${\overset{=}{\varepsilon }}_{g}=\left[\begin{array}{ccc} 0 & 0 & 0\\ 0 & \varepsilon_{YY} & 0\\ 0 & 0 & 0 \end{array}\right]\;\;\to \;\;{\overset{=}{\varepsilon }}_{l}=\left[\begin{array}{ccc} \varepsilon_{xx} & \varepsilon_{xy} & 0\\ \varepsilon_{xy} & \varepsilon_{yy} & 0\\ 0 & 0 & 0 \end{array}\right].$$

To overcome this issue, when the input strain field is not fully defined, elements should have a rectangular shape aligned with the input strain field direction. In this case, the transformation matrix applies only a rigid rotation of the different strain components, associating the right strain to the right direction and without changing their magnitude, as reported in Equation (6) and Figure 3b. In addition, the unique and direct correspondence between the strain directions in global and local coordinates allows a proper definition of the weighting coefficients $w_{\left(\cdot \right)}$, associating the right value (high or low coefficient) to each direction.

(6) $${\overset{=}{\varepsilon }}_{g}=\left[\begin{array}{ccc} 0 & 0 & 0\\ 0 & \varepsilon_{YY} & 0\\ 0 & 0 & 0 \end{array}\right]\;\;\to \;\;{\overset{=}{\varepsilon }}_{l}=\left[\begin{array}{ccc} \varepsilon_{xx} & 0 & 0\\ 0 & 0 & 0\\ 0 & 0 & 0 \end{array}\right]\;\;\mathrm{with}\;\;\varepsilon_{xx}=\varepsilon_{YY}$$

For the sake of completeness, when the input strain field is not fully defined, the iFEM minimization problem is also no more load independent. In particular, if the sensor network is not optimized for the particular case under analysis, considering all the possible loading conditions, the iFEM may lead to wrong full-field reconstructions. To limit this issue, sensors must accurately describe the structure’s strain field, in particular in the load direction, and input strain pre-extrapolation can increase the overall accuracy of the results, as described in [40,41]

### 2.3. Matrix Formulation

Once the input and numerical strain fields have been correctly defined, Equation (1) should be computed developing the Euclidean norms. In the most general case, considering an element covered by n strain sensors (measured or pre-extrapolated), the three contributions can be computed as:

(7) $$\|e\left(u^{i}\right)-e_{i}^{\varepsilon }{\|}^{2}=\frac{1}{n}\iint_{A_{i}}^{}\overset{n}{\underset{j=i}{\sum }}{\left(e{\left(u^{i}\right)}_{j}-e_{i,j}^{\varepsilon }\right)}^{2}dxdy\;\|k\left(u^{i}\right)-k_{i}^{\varepsilon }{\|}^{2}=\frac{{\left(2h\right)}^{2}}{n}\iint_{A_{i}}^{}\overset{n}{\underset{j=i}{\sum }}{\left(k{\left(u^{i}\right)}_{j}-k_{i,j}^{\varepsilon }\right)}^{2}dxdy\;\|g\left(u^{i}\right)-g_{i}^{\varepsilon }{\|}^{2}=\frac{1}{n}\iint_{A_{i}}^{}\sum_{j=i}^{n}{\left(g{\left(u^{i}\right)}_{j}-g_{i,j}^{\varepsilon }\right)}^{2}dxdy.$$

In the case an element is free from any strain input (both measured and pre-extrapolated), like the transverse shear, n is conventionally posed equal to one to avoid singularity and a small weighting coefficient $w_{\left(\cdot \right)}$ will be adopted to maintain the connectivity between elements. Finally, after the substitution of Equations (2) and (3) into Equation (1), the least-square functional of the *i*-th inverse element can be written as:

(8) $$\Phi_{i}\left(u^{i}\right)=u^{i^{T}}K^{i}u^{i}-2u^{i^{T}}f^{i}+\xi^{i}$$

in which:

(9) $$k^{i}=\iint_{A_{i}}^{}\left(w_{m}B^{m^{T}}B^{m}+w_{b}{\left(2h\right)}^{2}B^{b^{T}}B^{b}+w_{s}B^{s^{T}}B^{s}\right)dxdy\;f^{i}=\frac{1}{n}\iint_{A_{i}}^{}\overset{n}{\underset{j=1}{\sum }}\left(w_{m}B^{m^{T}}e_{i,j}^{\varepsilon }+w_{b}{\left(2h\right)}^{2}B^{b^{T}}k_{i,j}^{\varepsilon }+w_{s}B^{s^{T}}g_{i,j}^{\varepsilon }\right)dxdy\;\xi^{i}=\frac{1}{n}\iint_{A_{i}}^{}\sum_{j=1}^{n}\left(w_{m}e_{i,j}^{\varepsilon^{T}}e_{i,j}^{\varepsilon }+w_{b}{\left(2h\right)}^{2}k_{i,j}^{\varepsilon^{T}}k_{i,j}^{\varepsilon }+w_{s}g_{i,j}^{\varepsilon^{T}}g_{i,j}^{\varepsilon }\right)dxdy.$$

Finally, considering the contribution of the different elements with a standard assembly procedure, minimizing the error functional with respect to the displacement vector in global coordinated (i.e., ∂Φ/∂U =0), and applying problem-specific boundary conditions to avoid any free motion, the unconstrained dof $U_{F}$ can be computed as:

(10) $$U_{F}=K_{FF}^{-1}\cdot F_{F}$$

where $K_{FF}^{}$ is the stiffness-like matrix associated with the unconstrained dof linking displacements and strains, while $F_{F}$ is a vector function of the input strains. Notice that, considering a structure with a given sensor network, the stiffness matrix does not depend on the input field and thus can be computed and inverted just once. While only the vector $F_{F}$ has to be updated with the new strain measurements acquired in each time instant, making the final computation very efficient and suitable for real-time applications.

### 2.4. Superimposition of the Effects with iFEM

Model definition is one of the most challenging tasks for both direct and inverse FEM, in particular when dealing with non-trivial geometries and boundary conditions. The linear superimposition of effects with iFEM has been still introduced in [37], weighting the contribution of different iFEM elementary models to better approximate the reference displacement field. In particular, the elementary models must have the same geometrical discretization, i.e., the same nodes and elements, while different boundary conditions can be applied to better model non-trivial constraints. Finally, the displacement contribution of each elementary model is weighted with the following equation, computing the displacement field $U_{c}$ of the combined model.

(11) $$U_{c}=\sum_{i=1}^{N_{m}}U_{i}\cdot \alpha_{i}\;\;\mathrm{with}\;\;\sum_{i=1}^{N_{m}}\alpha_{i}=1$$

where $N_{m}$ is the number of elementary models exploited, $U_{i}$ the nodal displacements of each model, and $\alpha_{i}$ the related weighting coefficient.

The fundamental aspect of this approach is the definition of the weighting coefficients $\alpha_{i}$, which must guarantee the coherence between the model and the real structure’s displacement field. This is ensured by the minimization of the following error function, defined as the comparison between the reference displacement field $U_{ref}$ and the reconstructed combined model $U_{c}$, which is a function of the unknown weights.

(12) $$E_{r}={\left(U_{ref}-U_{c}\right)}^{T}\cdot \left(U_{ref}-U_{c}\right)$$

The reference displacement field can be defined with both high fidelity direct FEM or experimental displacement measurements on the structure. However, when leveraging on direct FEM, the error can account for the magnitude displacement of all the structural nodes, performing a global error minimization.

## 3. Experimental Tests

**Figure 4:**
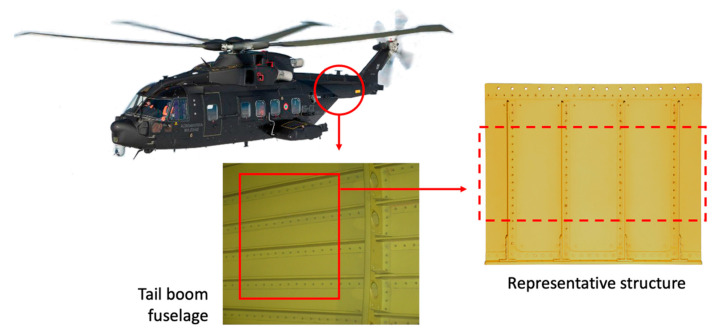
Helicopter with representative structure.

The experimental tests are carried out on a representative structure of the tail boom of a medium-weight helicopter’s fuselage, as described in Figure 4, to verify the shape sensing capability of a permanently installed sensor network. The structure and the sensor network are described in Section 3.1, then the experimental test rig is detailed in Section 3.2.

### 3.1. Aeronautical Structure and Sensors Network

**Figure 5:**
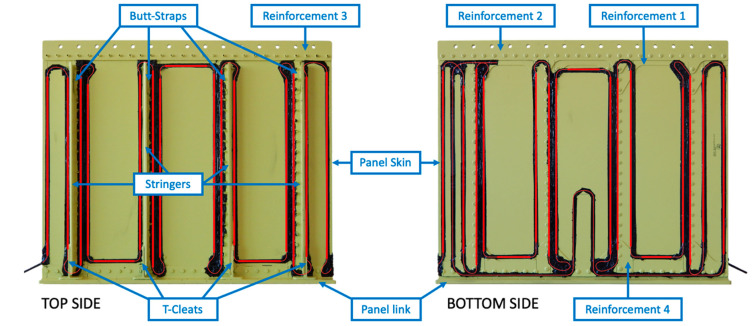
Aeronautical structure with OBR optical fiber (in red) and indications of the main parts.

The panel structure consists of different aluminum sheets joined together by rivets and reinforced by four stringers. The overall dimensions are 600 × 500 mm with a stringers’ depth of 25.7 mm. Different aluminum alloys and thicknesses are adopted for the different parts to correctly reproduce the behavior of the parent structure, as shown in Figure 5 and reported in Table 1 and Table 2. In particular, the lower side of the panel is realized by a panel link to obtain rigid constrains to the ground through bolt connections, while the upper side contains some reinforcements and holes for its connection with an actuator. As highlighted in Figure 4, only the central region of the structure, i.e., the panel skin and the stringers, is representative of the rear fuselage, while the other reinforcements are specifically designed to constraint the specimen and provide a correct load transfer.

Strains are acquired with an Optical Backscatter Reflectometer (OBR) connected to 15 m of optical fiber glued on both sides of the structure with an almost symmetric pattern, as described in Figure 5. Hereafter, the internal and external sides of the panel are referred to as top and bottom sides, respectively, to correlate with the element configuration in Figure 2. The fiber’s pattern is optimized to measure the strain field on the panel skin and stringers, allowing a better structure’s shape sensing. This fiber technology provides an almost continuous strain measure (every 2.5 mm) along its whole length. However, only the straight segments aligned or transversal to the global reference system are exploited as input, as highlighted in bold red. For the sake of clarity, most of the measurement points available are aligned with the load direction, few strain data are transversal to the load direction, and no sensors define the shear strain component.

### 3.2. Test Rig

**Figure 6:**
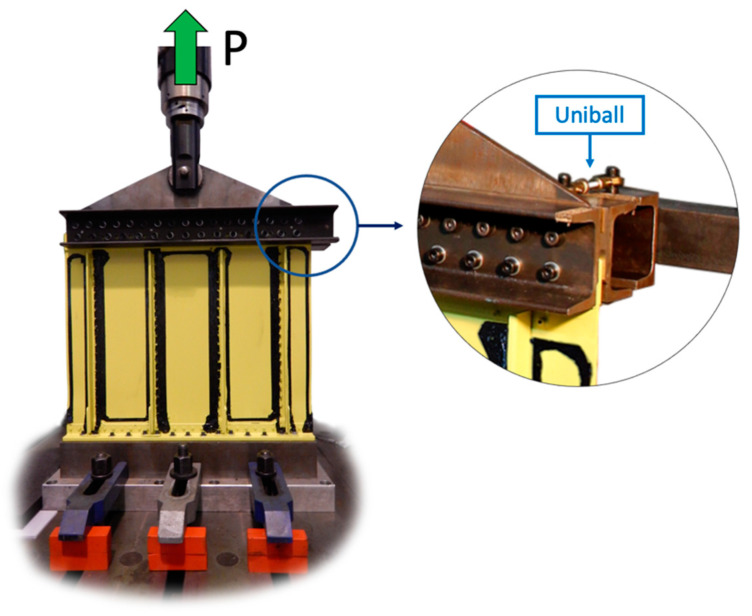
Experimental test rig.

The panel is constrained to the ground on its lower side and connected to an MTS 244.11 hydraulic actuator on the upper side, as reported in Figure 6.

The lower side of the structure is firstly constrained to a steel basement through bolt connections, then it is fixed to the laboratory’s floor by means of other 6 (3 per side) bolt connections.

The upper side of the aeronautical structure is linked to a high stiffness steel triangle with two C-shape beams (one per side) and bolt connections. Then, a hydraulic actuator is connected to the upper side of the linking triangle with a pin to apply a monoaxial vertical load. In addition, the C-shape beam profile on the structure’s bottom side is connected to the ground with two Uniballs to constrain the system in the out-of-plane direction.

The hydraulic actuator applies a static tension load P of 35 kN in the vertical direction, representative of the helicopter’s parent structure operative conditions. Strains are acquired through the LUNA ODISI-B system at a sampling frequency of 3 Hz, then different time instants are averaged together to increase the data accuracy. Finally, the actuator’s displacement is stored as a reference, although this also contains the compliance of the entire test rig.

## 4. Numerical Models

A detailed direct FE model of the aeronautical panel is first developed to numerically simulate the structure’s behavior in a virtual environment and obtain its full displacement and strain fields. Then, different inverse FEM models are developed to assess the shape sensing capability with both numerically simulated and experimentally acquired input strain data.

### 4.1. Direct FEM Model

**Figure 7:**
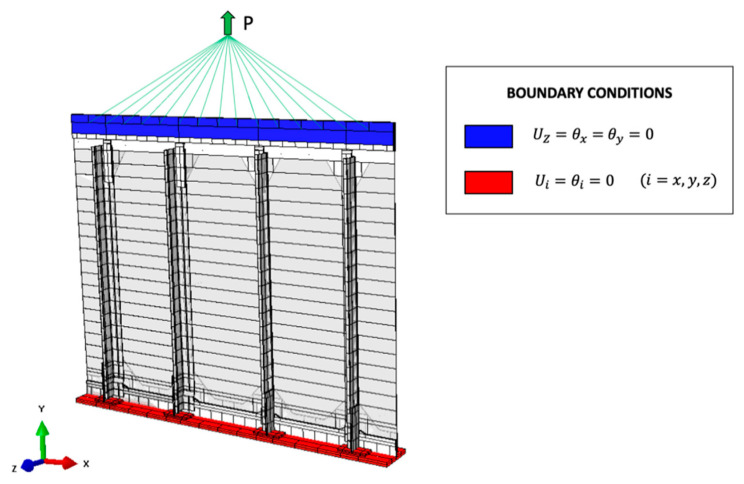
Model for the high-fidelity direct FEM.

The direct FE model is a high-fidelity reproduction of the physical structure, where the different parts are modeled independently and then connected together with interactions representative of the rivets’ behavior. In particular, a spring connection simulates the presence of each rivet and the different shells are in contact with each other with a frictionless hard contact. The applied boundary conditions replicate the physical structure’s behavior, with a clamp and a slider on the lower and upper sides, respectively, as reported in Figure 7. A concentrated load of 35 kN is applied on a reference point, mimicking the actuator’s connection to the infinitely rigid steel triangle, and then transferred to the structure with a kinematic coupling. Finally, the overall structure is discretized with 55008 S8R shell elements with quadratic formulation.

### 4.2. Inverse FEM Models

A fundamental requirement of the iFEM algorithm is the model definition with its boundary conditions. The aeronautical panel under analysis represents a complex geometry composed of different parts linked together by rivets, as already described in Section 3.1. The related iFEM model definition is non-trivial, in particular when leveraging on a limited number of strain sensors. For this reason, two different iFEM models are proposed: (i) A *complete* model, presented in Section 4.2.1, is only devoted to a numerical study, allowing the input strain field definition for the entire structure, (ii) a *simplified* model, Section 4.2.2, contains several simplifications to account for the experimental sensor network.

#### 4.2.1. Complete iFEM Model

**Figure 8:**
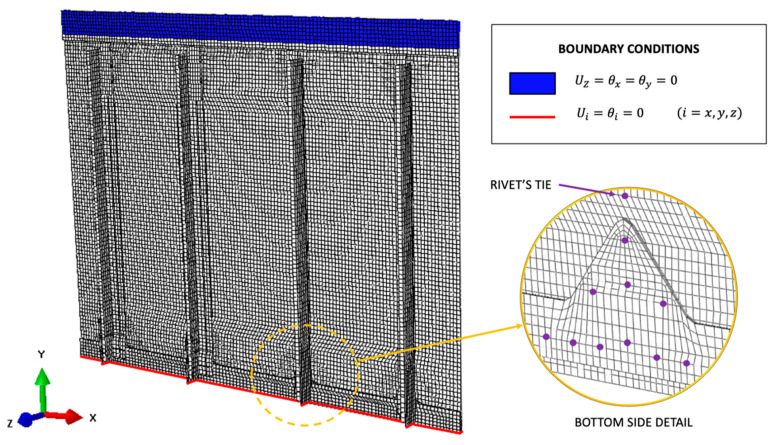
Complete inverse Finite Element Method (iFEM) model.

The complete model is a high-fidelity reproduction of the real structure, containing almost the same geometry of the direct FEM model. In particular, the different aluminum skins are modeled as independent shells maintaining the same geometry of the direct FEM. Only the panel link to the external test rig is simplified to remove unnecessary elements on the constrained regions. The main simplification regards the rivets interaction, which is no more modeled as a spring but with a rigid TIE between all the nodes interested by the same rivet. Also, the hard contact interaction between the different shells is no more present since the iFEM algorithm is only based on strain–displacement relations. The input strain field is fully defined on both sides of each element, ensuring the best reconstruction: This represents the ideal case in which each inverse element contains a strain gauge rosette on its centroid positions both on the top and bottom sides, also where the shell’s surface is in contact with another structural element. Obviously, this cannot be easily implemented in a real scenario and has to be regarded as a preliminary methodology assessment, where the iFEM is fed with numerically simulated strains computed with the direct FEM to assess the iFEM shape sensing capability in a complex scenario. The iFEM includes 23392 iQS4 inverse elements and considers the same boundary conditions as adopted in the direct FEM simulation, as reported in Figure 8.

This model cannot be exploited in the case of the experimental sensor network introduced in Section 3.1 for two main reasons related to the input strain field definition. The first reason is related to the limitation in the number of strain measurements. In particular, the iFEM requires the input strain definition on both sides of the elements to correctly decouple the membrane and the bending strain components. However, this is not possible where two or more shells are joint together by rivets, such as in correspondence of stringers, where sensors are applied on the top of stringers and the bottom side of the skin panel. Furthermore, many reinforcements shells are free from any strain sensor, making the input strain field definition even more challenging. The second reason is related to the presence of non-rectangular elements with a partial definition of the input strain field, leading to numerical errors, as described in Section 2.2. The sensor network in Section 3.1 only allows a full definition of $\varepsilon_{XX}$ and $\varepsilon_{YY}$ in the control region highlighted in Figure 4, characterized by rectangular element and where strains can be easily pre-extrapolated with the SEA in places where physical sensors are not available, although no definition of $\varepsilon_{XY}$ is possible.

#### 4.2.2. Simplified iFEM Model

**Figure 9:**
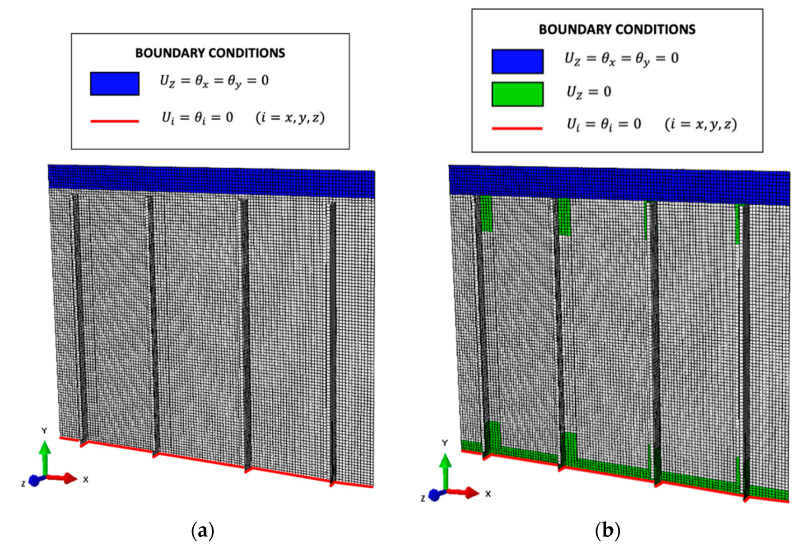
Simplified iFEM models: (**a**) Model 1 with base boundary conditions; (**b**) Model 2 with high stiffness boundary conditions.

To overcome the issues presented in Section 4.2.1, the simplified iFEM model considers different geometrical simplifications and adopts only inverse elements with a rectangular shape, as shown in Figure 9. Since shape reconstruction is based on the strain measurements acquired on the skin plate and on the stringers (Figure 5), the boundary reinforcements are removed from the model decreasing the overall complexity. The resulting geometry has a constant thickness equal to 0.8 mm (skin plate’s thickness), except in correspondence of the stringers, where the stringers and the skin are merged into a single shell with an overall thickness equal to the sum of the two (2.1 mm), allowing the input strain definition on both sides of the elements. However, the iFEM structure obtained has a lower stiffness than the real one due to the elimination of reinforcements. Even though the constraints imposed by the reinforcements have a specific stiffness which is not easy to be estimated and reproduced in the iFEM model, this can be taken into consideration by a linear superimposition of the multiple models with different boundary conditions.

Thus, the geometry is discretized into a mesh of 14078 iQS4 inverse elements, having the same average element’s size as the complete iFEM model, and considering two different sets of boundary conditions. The first model (Figure 9a) contains the same boundary conditions as the complete iFEM model (and the direct FEM), underestimating the overall stiffness of the structure as previously described. In order to compensate for this effect, the second model (Figure 9b) overestimates the overall stiffness constraining different additional regions. In particular, the out-of-plane displacement of the T-Cleats, of some stringer’s regions, and of the Butt-Straps are set to zero. Finally, both simplified iFEM models are computed independently and then their linear superimposition is applied as in Section 2.4.

The simplified iFEM model is firstly assessed with numerically simulated strains to compute the weighting coefficients $\alpha_{i}$ and then it is fed with the experimental measurements to verify the overall approach. The numerical strains extracted from the direct FEM mimic the experimental sensor network and consist of monoaxial strain measurements associated with the centroid of each inverse element crossed by the optical fiber. Then, the displacement field results of the two simplified models are combined to compute the weighting coefficients $\alpha_{i}$ minimizing Equation (12) with respect to the direct FEM displacement field in the control region highlighted in Figure 4. Notice that, since the input strain field is not fully defined on the entire model, a different sensor network would lead to a different set of weighting coefficients. Then, in the case of experimental measurements, a non-perfect match of sensor position in the two sides of the shell structure will induce errors in the membrane and bending strain components decoupling, which can be mitigated if multiple measurement points falling within an inverse element are averaged and conventionally applied at the element centroid, although for limited mesh size. In this specific case, the simplified iFEM model contains elements with a size of 5 mm, thus in each element 2 strain measurements are averaged into a single value with a negligible overall approximation on the shape sensing results. The mesh size has been defined based on a sensitivity analysis, which is not reported for brevity.

## 5. Results and Discussion

The direct FEM results are firstly reported in Section 5.1, setting the structure’s target displacement field, then, the iFEM reconstructions based on the complete model is described in Section 5.2. Since the complete model cannot be exploited with the experimental sensor network, a simplified iFEM model is developed exploiting geometrical simplifications and the superimposition of the effects. In particular, Section 5.3 presents the results obtained with the simplified model and computes the weighting coefficients of the different elementary models. Then, Section 5.4 collects the results obtained with the simplified iFEM model and numerical strain measurements affected by noise, to assess its shape sensing capability in a more realistic scenario. Section 5.5 provides a comparison and a critical analysis of the two iFEM models (complete and simplified versions) with respect to the direct FEM. Finally, the experimentally acquired strains are given as input to the simplified iFEM model and the results are described in Section 5.6.

### 5.1. Direct FEM Results

**Figure 10:**
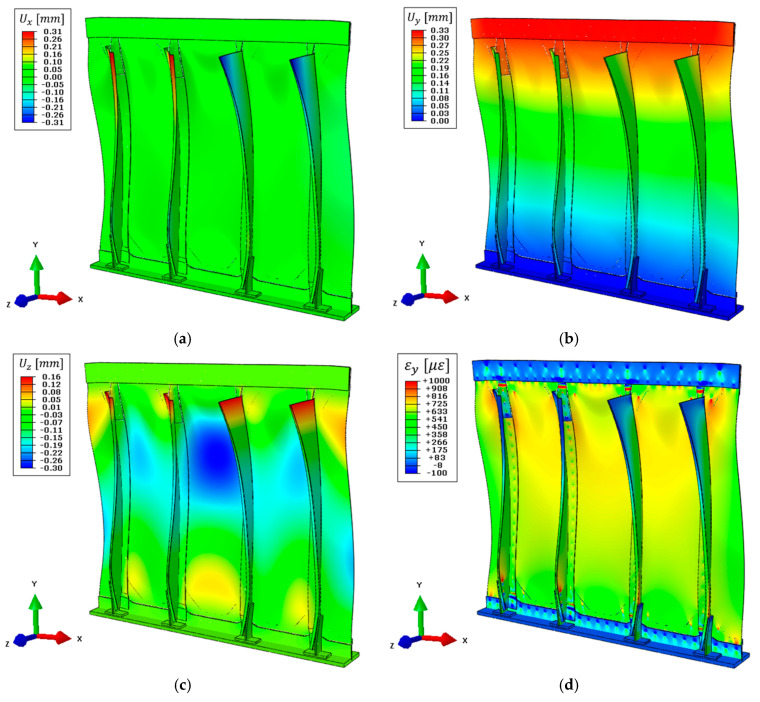
Direct FEM results, deformed shape with scale factor 100: (**a**) Displacement field on the x-direction, (**b**) displacement field on the y-direction, (**c**) displacement field on the z-direction, and (**d**) strain field on the y-direction on the top side of the structure.

The full displacement field obtained with the high-fidelity direct FEM previously introduced in Section 3.1 is reported in Figure 10. The structure is subjected to a constant tension load in the y-direction, which is reflected in a maximum displacement of 0.33 mm on the upper side (Figure 10b). Then, the complex structure’s geometry induces an out-of-plane displacement field on the panel skin of 0.30 mm on its central region (Figure 10c) and a maximum stringer’s displacement of 0.31 mm on the x-direction (Figure 10a). The presented displacement field is assumed as the reference target value for the numerical iFEM reconstructions and the computation of the coefficients for boundary effect superimposition.

The direct FEM is also exploited to compute the full strain field on the whole structure which is given as input to both the complete and the simplified iFEM models. In particular, the strain field on the top side of the structure along the load direction (vertical in the figure) is reported in Figure 10d.

### 5.2. Numerical iFEM Results with Complete Model

The complete iFEM model is fed with numerically simulated input strains, which are extracted from the direct FEM simulation. In each inverse element, a strain gauge rosette is located on the element’s centroid on both sides of the shell, for a full definition of the input strain field.

**Figure 11:**
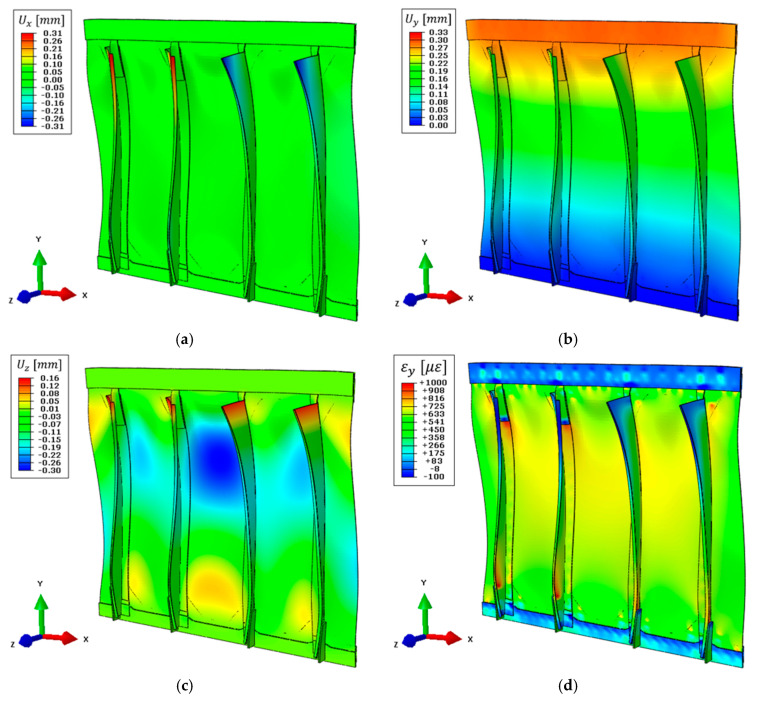
Complete iFEM model results with full definition of the input strain field, deformed shape with scale factor 100: (**a**) Displacement field on the x-direction, (**b**) displacement field on the y-direction, (**c**) displacement field on the z-direction, (**d**) strain field on the y-direction on the top side of the structure.

The complete iFEM results are reported in Figure 11, where displacements are in good agreement with the direct FEM (Figure 10). For an easier interpretation and comparison of the results, displacements and strain shown in Figure 11 are represented with the same color scale range as Figure 10. In particular, the out-of-plane displacement field (Figure 11c) and the displacements along the x-direction (Figure 11a) almost perfectly match with the direct FEM, while a limited error is present along the vertical direction (Figure 11b). The latter gives a maximum displacement of 0.30 mm, underestimating the target value of about 9% mainly due to the approximation introduced with the rivet’s modeling. In particular, the iFEM model considers a rigid link between all nodes interested by each rivet, resulting in a stiffer model than the reference direct FEM.

As for any direct FEM, the iFEM strains are computed from nodal displacements. The strain component aligned with the load direction is reported in Figure 11d. In this case, the results are qualitatively in good agreement with the direct FEM. However, a more detailed comparison will be provided in Section 5.5.

The complete iFEM model offers a high-fidelity representation of the physical structure, although sensor network limitations prevent its application to the experimental case study.

### 5.3. Numerical iFEM Results with the Simplified Model

The simplified model is given by the linear superimposition of two elementary models, as described in Section 2.4. First, displacements are computed for the two elementary models with a sensor network that mimics the experimental one. The iFEM mesh elements crossed by the optical fiber are associated with a monoaxial input strain measure, extracted from the direct FEM, simulating the overall experimental input field, including positions on the skin and stringers, as shown in Figure 5, while SEA pre-extrapolation ensures the input strain field definition for those elements where physical sensors are not available. This procedure computes two different sets of nodal displacements, not reported here for the sake of brevity, related to the two simplified models, respectively.

The displacement fields of the two elementary models are combined with weighting coefficients that minimize Equation (12). Results are reported in Table 3, where, as expected, the combined model is an intermediate condition between the simplified model 1 (low stiffness, accounting for 30.4%) and the simplified model 2 (high stiffness, accounting for 69.6%). Specifically, the error function of Equation (12) is an index of the global matching between the iFEM displacement results and the reference direct FEM values, thus it can be used to further appreciate the improvement of model accuracy. In particular, the global error obtained with the combined model offers a lower error than both elementary models, as reported in Table 4. This verifies the effectiveness of the approach adopted, where the reduced stiffness due to removal of the reinforcements is restored by superposition of multiple models.

**Figure 12:**
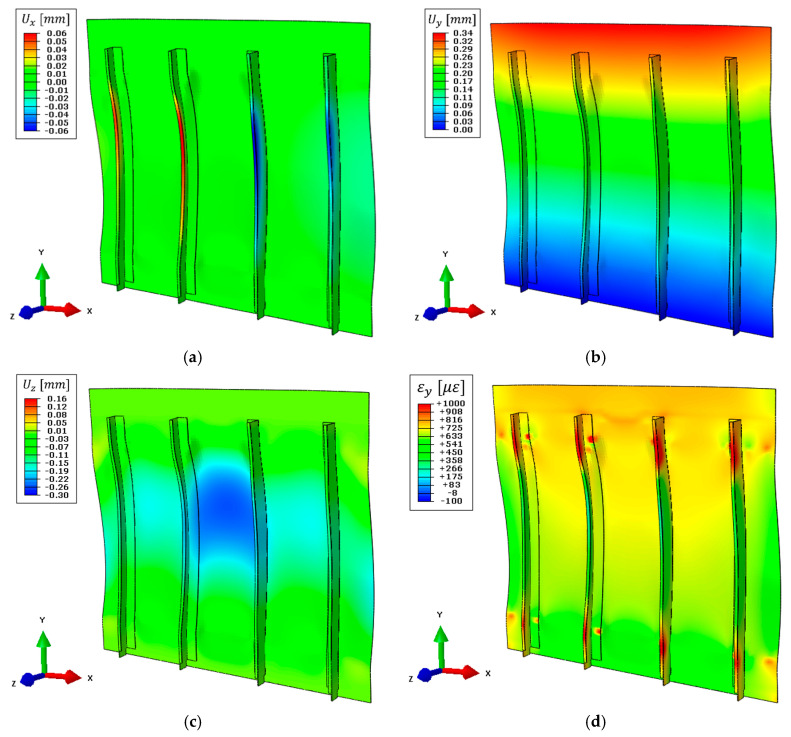
Simplified iFEM model results with experimental sensor network and numerically simulated input strain field, deformed shape with scale factor 100: (**a**) Displacement field on the x-direction, (**b**) displacement field on the y-direction, (**c**) displacement field on the z-direction, (**d**) strain field on the y-direction on the top side of the structure.

The nodal displacements of the combined model are computed with Equation (11) and reported in Figure 12, where the same color scale range as in Figure 10 is adopted, when possible, for easier comparison between the two. The maximum vertical displacement obtained is 0.34 mm (Figure 12b), slightly overestimating (+3%) the target value, confirming the effectiveness of the pre-extrapolation along the vertical direction. Then, the out-of-plane displacement (Figure 12c) still describes the overall structure’s behavior, however with higher errors due to the limited information available as input. For example, the displacement in the central region of the panel is 0.27 mm, underestimating the target value by −10%, ensuring an acceptable reconstruction also far from the input sensors. Finally, the limited strain information in x-direction is responsible for the error in describing the stringers’ displacement field, which is limited to 0.06 mm as the maximum value (Figure 12a).

Then, strains are computed on the whole structure and its component along the load direction is reported in Figure 12d. Strains on the central region of the structure (where the sensor network is installed) are still coherent with the reference direct FEM and further comparisons will be provided in Section 5.5.

It can be concluded the combination of simplified iFEM models achieved a sufficiently accurate displacement reconstruction at a low computational burden, even in presence of a reduced sensor network.

### 5.4. Numerical iFEM Results with the Simplified Model and Noise Contribution

**Figure 13:**
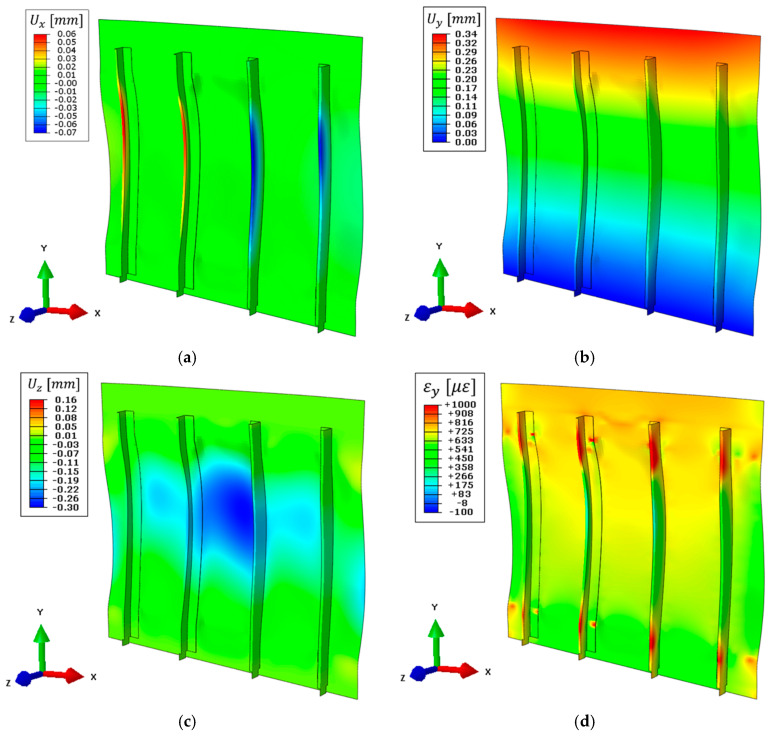
Simplified iFEM model results with experimental sensor network and numerically simulated input strain field affected by noise, deformed shape with scale factor 100: (**a**) Displacement field on the x-direction, (**b**) displacement field on the y-direction, (**c**) displacement field on the z-direction, (**d**) strain field on the y-direction on the top side of the structure.

Once the model’s weighting coefficients have been computed in a controlled environment, considering only the models’ contribution, the combined model is assessed in the presence of noise since this is not always erasable in real-time applications. In particular, the noise level introduced mimics the optical fiber adopted for the experimental tests, having a Gaussian distribution with an average standard deviation of 10 με for the different measurement points. Numerical strains computed with the direct FEM have been affected by noise to compute the two elementary models with iFEM, then, the combined model is obtained with the weighting coefficients reported in Table 3. Displacement and strain field results are reported in Figure 13, where the same color scale range as in Figure 10 is adopted, when possible. Results are qualitatively close to the same simulation without noise (Figure 12); however, the noise contribution is reflected into a non-symmetric displacement field. In particular, this can be appreciated in Figure 13a for the displacement field along the x-direction and in Figure 13c for the out-of-plane displacement field. Furthermore, the error function of Equation (12) is now increased to $16.25\;{\mathrm{mm}}^{2}$, with respect to $15.52\;{\mathrm{mm}}^{2}$ without noise (Table 4), confirming a small decrease in the iFEM performances. Nevertheless, the overall iFEM reconstruction is assessed also in presence of noise and more detailed comparisons for both the displacement and the strain fields will be provided in Section 5.5.

### 5.5. Comparison between iFEM Results

**Figure 14:**
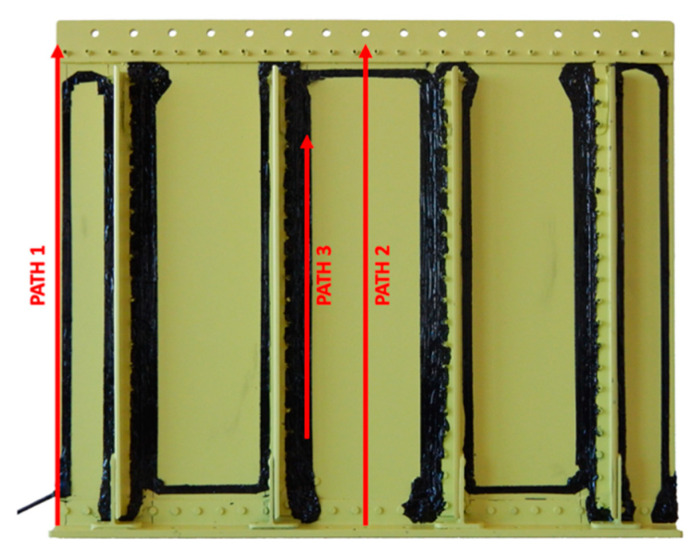
Extraction paths for results comparison.

A joint comparison of the iFEM displacements obtained in Section 5.2 (for the complete model) and Section 5.3 and Section 5.4 (for the combination of simplified models, herein referred to as simplified model) with the direct FEM displacements shown in Section 5.1 is provided in this section. For the sake of simplicity, the comparison is limited to three vertical paths: (i) On one side of the panel, (ii) on the central panel bay, and (iii) in correspondence of an optical fiber located on the panel skin, as highlighted in Figure 14.

**Figure 15:**
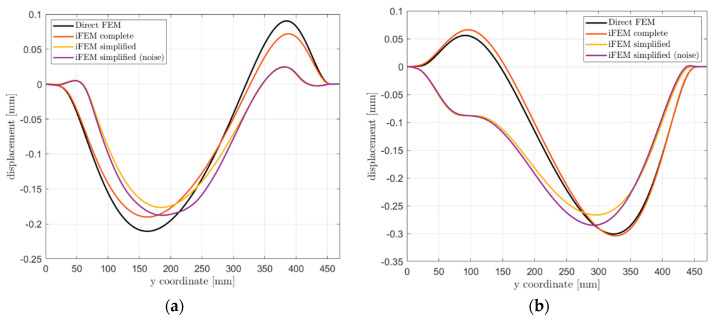
Out-of-plane displacement $U_{z}$ comparison: (a) Path 1; (b) Path 2.

The first comparison is related to the out-of-plane displacement along paths 1 and 2, as shown in Figure 15. The complete iFEM model almost perfectly reproduces the reference displacement field, while higher errors are present in the simplified one due to the limited number of sensors. In particular, sensors are not able to fully describe the structure’s strain field, resulting in a different final deformed shape. Strain pre-extrapolation with SEA mitigates this problem, however, few measurement points are available in the x-direction and no sensors measure the shear strain component, limiting the overall shape sensing capability. Noise contribution additionally affects the displacement field, producing slightly different results far from constraints. Finally, Table 5 reports a comparison between the displacement fields computed with the different models for a more synthetic overview of the reconstruction accuracy. Notice that, the maximum displacement components can be referred to different points of the structure, as also visible in Figure 15.

**Figure 16:**
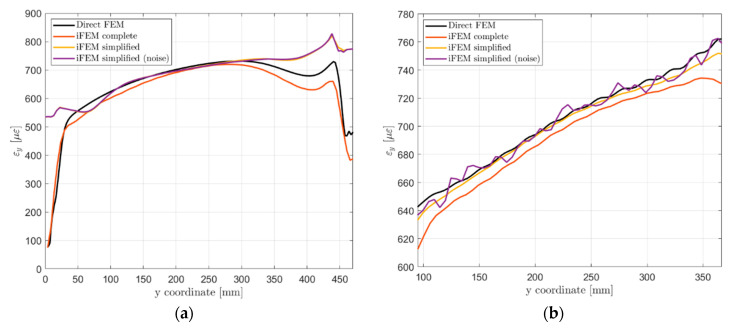
Vertical strain component $\varepsilon_{y}$ comparison on the top side of the structure: (a) Path 2; (b) Path 3.

A second comparison in Figure 16 takes into account the strain component on the load direction along paths 2 and 3. Considering the path in the central bay (path 2, Figure 16a) the complete iFEM model well reproduces the reference strain field due to the input provided for all elements and the high-fidelity geometry description. The simplified iFEM model well describes the strain field in the central region of the panel, confirming the effectiveness of the iFEM reconstruction far from the input sensors. Only the upper and lower parts of the specimen exhibit a different strain behavior due to the different geometry and boundary conditions adopted. Results affected by sensors’ noise are almost perfectly overlapped to the ones without noise since the path under analysis is far from input sensors. Differently from the previous case, the simplified iFEM model (without noise) better predicts the strain field along path 3 than the complete iFEM model (Figure 16b). The reason for this potentially anomalous behavior lies in the iFEM optimization process, which relies on a set of weighting coefficients $w_{\left(\cdot \right)}$, ensuring a proper reconstruction also when an input is not provided in all elements. The complete model leverages on a full definition of the input field, thus all elements have the same unitary weight in the minimization process. On the contrary, considering the simplified model, elements along path 3 have high weighting coefficients with respect to the other elements not crossed by the optical fiber, giving more importance to potential reconstruction errors near the sensors. For the same reason, the simplified model affected by sensors’ noise produced a strain field which is locally high affected by this contribution. Direct FEM strain field along path 3 (Figure 16b) also exhibits a sinusoidal component given by the rivets’ load transfer with the adjacent stringer and this is also partially reproduced by the iFEM simulations.

### 5.6. iFEM Results with Experimental Input Strain

The combined simplified iFEM model, leveraging on the same coefficients reported in Table 3, is now used to process experimental strains, acquired on the test rig described in Section 3.2.

**Figure 17:**
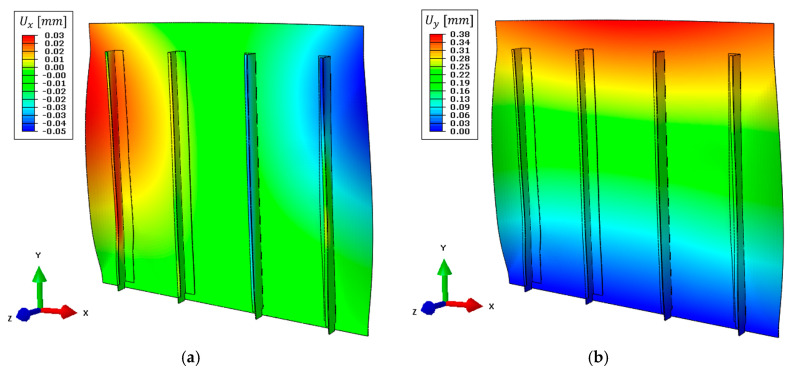

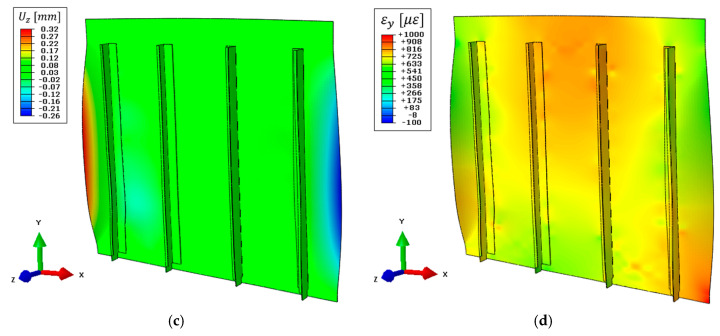
Simplified iFEM model results with experimental input strain measurements, deformed shape with scale factor 100: (**a**) Displacement field on the x-direction, (**b**) displacement field on the y-direction, (**c**) displacement field on the z-direction, (**d**) strain field on the y-direction on the top side of the structure.

Experimental strain measures acquired with the LUNA ODISI-B system were highly influenced by several noisy vibrations and disturbances coming from the environment which hampered the quality of the acquired strains, affecting the iFEM shape sensing performance. In order to limit this effect, different time instants have been averaged together; however, some contributions could not be eliminated with this procedure. For this reason, the weighting coefficients $w_{\left(\cdot \right)}$ associated with elements with pre-extrapolated strain have been increased to 0.1 (instead of $10^{-5}$ as in Section 5.3 and Section 5.4), increasing the influence of the smooth strain field computed with the SEA. In particular, the latter limits random deflections of the skin panel, which would be induced by disturbances contribution on the bending strain component’s computation. Finally, displacement results are reported in Figure 17, where these effects can be qualitatively observed. In particular, the maximum vertical displacement obtained is 0.38 mm (Figure 17b), the displacement field along the x-direction (Figure 17a) is not able the describe the stringer’s movement due to the sensor network’s limitations (as described in Section 5.3), and the out-of-plane displacement (Figure 17c) does not describe the actual panel’s movement due to the problems previously described.

As for displacements, no direct measure of the panel displacements is available, however, the displacement of the actuator (0.64 mm), including the compliance of the test rig (which is only partially estimated in about 0.11 mm), is higher than the displacement calculated by the iFEM for the panel specimen only, as expected.

**Figure 18:**
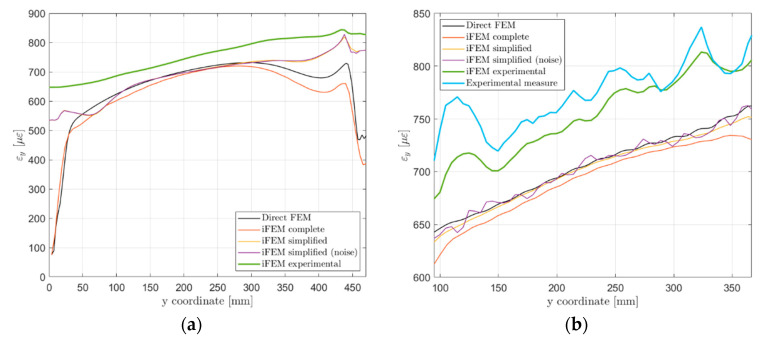
Vertical strain component $\varepsilon_{y}$ comparison on the top side of the structure with experimental results: (**a**) Path 2; (**b**) Path 3.

The iFEM accuracy can be evaluated also in terms of strain field reconstruction, comparing the iFEM strain results with the experimentally acquired values. The strain component along the load direction is computed for the entire structure and shown in Figure 17d. Then, for an easier interpretation and comparison of the results, the same curves shown in Figure 16 are reported in Figure 18 with the addition of the experimental results. Figure 18b displays the strain field along path 3, crossed by the optical fiber. Experimental strains acquired by the optical fiber have a higher value than those simulated with the direct FEM, contributing to the bigger vertical displacement computed by iFEM. Then, strains computed with iFEM displacements follow the experimental input field, which is highly influenced by noise as previously described. Finally, considering the strain results along path 2 (Figure 18a), the iFEM confirms its ability to predict the strain field also far from input sensors since the global behavior is coherent with the related numerical simulation, just a bias is present due to the higher sensor’s input. The global higher magnitude of the sensor’s input strain field can be attributed to the experimental errors and noise present during the test; however, these do not affect the validity of the whole procedure and the obtained results.

The iFEM was demonstrated to be suitable for shape and strain sensing also in real complex scenarios. However, the reconstruction’s accuracy is subordinated to the quality of the input strain measurements.

## 6. Conclusions

In this work, the inverse Finite Element Method (iFEM) is exploited for the shape sensing of a complex aluminum aeronautical structure, which is representative of the tail boom of a medium-weight helicopter’s fuselage, subjected to a tension load in a laboratory experiment and resulting in complex displacement and strain fields.

The structure’s displacement field is firstly simulated with a direct FE analysis, leveraging on a high-fidelity model to reproduce the physical behavior of the specimen. These results are passed as input to different iFEM models for testing the methodology in a virtual environment.

The development of a high-fidelity iFEM model confirmed the algorithm’s shape sensing capability also in the presence of complex displacement and strain fields, although based on a full definition of the input strain field. Nevertheless, experimental applications often rely on a limited number of strain sensors, depending on the available acquisition system and physical constraints imposed by the structure itself. Thus, the high-fidelity iFEM model may not be exploitable in real application scenarios, requiring the definition of simplified iFEM models. Geometrical simplifications revealed to be a valuable tool in iFEM applications, reproducing the structure’s behavior at a low computational burden. In particular, non-relevant parts can be simplified and their original stiffness is restored by proper tuning of the constraints, where a linear superimposition of different boundary conditions can account for the actual structural stiffness of the components. The weighting coefficients required by the superimposition of the effects can be computed minimizing an error function to guarantee the matching of the structure’s displacement field with respect to direct FEM reference values. Then, the overall methodology has been confirmed by experimentally acquired strain measurements in presence of a high level of uncertainty. It has been demonstrated that the simplified iFEM model correctly computed the strain field also far from input sensors and in presence of a complex strain field, confirming the effectiveness of the approach.

Future activities of the authors will be devoted to the damage detection on the analyzed aeronautical structure, to further extend the iFEM capability in view of real on-board applications.

## Figures and Tables

**Table 1:** Material and thickness of the structure’s elements.

Part	Material	Thickness [mm]
Panel Skin	Al2024	0.8
Stringers	Al7475	1.3
Reinforcement 1	Al2024	1.0
Reinforcement 2	Al2024	1.6
Reinforcement 3	Al2024	1.6
Reinforcement 4	Al2024	1.6
T-Cleats	Al8090	4.0
Butt-Straps	Al2024	1.6
Panel link	Al7075	3.0

**Table 2:** Material properties.

Material	*E* [MPa]	*ν*
Al2024	72,400	0.33
Al7075	71,000	0.33
Al7475	71,000	0.33
Al8090	77,000	0.33

**Table 3:** Combined model’s weighting coefficients.

Model	*α*
Simplified iFEM model 1	0.304
Simplified iFEM model 2	0.696

**Table 4:** Error functional of different simplified iFEM models.

Model	*E_r_* [mm^2^]
Simplified iFEM model 1	29.36
Simplified iFEM model 2	18.15
iFEM combined model	15.52

**Table 5:** Comparison between maximum displacement results.

Model	$U_{x,max}\;\left[\mathrm{mm}\right]$	$U_{y,max}\;\left[\mathrm{mm}\right]$	$U_{z,max}\;\left[\mathrm{mm}\right]$	$U_{z,min}\;\left[\mathrm{mm}\right]$
Direct FEM (reference)	0.31	0.33	0.16	−0.30
Complete iFEM	0.31	0.30	0.15	−0.30
Simplified iFEM	0.06	0.34	0.02	−0.27
Simplified iFEM (noise)	0.06	0.34	0.03	−0.30

## Data Availability

Not applicable.

## Appendix A

The transformation matrices for the element’s dof and the sensors’ strain are derived hereafter.

The local reference system within each inverse element starts from the centroid’s definition, in which it is centered. In particular, considering an inverse element, the nodal coordinates $X_{q}$ in the global reference system are defined as

(A1) $$X_{q}=\left\{\begin{array}{c} X_{q}\\ Y_{q}\\ Z_{q} \end{array}\right\}\;\mathrm{with}\;q=1,\;2,\;3,\;4.$$

The local reference system is defined by the versors n, p, l, defining the normal direction and the two in-plane directions respectively.

(A2) $$n=\frac{X_{31}\wedge X_{42}}{\left\|X_{31}\wedge X_{42}\right\|}\;p=\frac{X_{31}+X_{42}}{\left\|X_{31}+X_{42}\right\|}\;l=p\wedge n$$

where the symbol ∧ indicates a vectorial product and $X_{ij}=X_{i}-X_{j}$. Then, the element’s centroid is defined, in global coordinates, with the following equation

(A3) $$C=\frac{\sum_{\alpha =1}^{4}c_{\alpha }\cdot d_{\alpha }}{\sum_{\alpha =1}^{4}d_{\alpha }}$$

where $c_{\alpha }$ is an edge mid-point and $d_{\alpha }$ the length of the same edge, which are defined as

(A4) $$c_{\alpha }=\frac{X_{\beta }+X_{\alpha }}{2}\;d_{\alpha }=\|X_{\beta }-X_{\alpha }\|$$

with the cyclic permutation α=1, 2, 3, 4 and β=2, 3, 4, 1 on the nodal coordinates X.

Finally, the strain’s transformation matrix from the global to the local reference system is defined as

(A5) $$T={\left[\begin{array}{ccc} l^{T} & p^{T} & n^{T} \end{array}\right]}^{T}$$

while the nodal dof transformation from the global reference system to the local one is defined as

(A6) $$u^{i}=T^{e}\cdot U^{i}$$

with

(A7) $$T^{e}=\left[\begin{array}{cccccccc} T & 0 & 0 & 0 & 0 & 0 & 0 & 0\\ 0 & T & 0 & 0 & 0 & 0 & 0 & 0\\ 0 & 0 & T & 0 & 0 & 0 & 0 & 0\\ 0 & 0 & 0 & T & 0 & 0 & 0 & 0\\ 0 & 0 & 0 & 0 & T & 0 & 0 & 0\\ 0 & 0 & 0 & 0 & 0 & T & 0 & 0\\ 0 & 0 & 0 & 0 & 0 & 0 & T & 0\\ 0 & 0 & 0 & 0 & 0 & 0 & 0 & T \end{array}\right]$$

where 0 is a 3 × 3 zero matrix. Note that $T^{e}$ is a 24 × 24 matrix, since each iQS4 element has 24 dof (3 translations and 3 rotations for each node).
